# The Teeth of Recruits

**Published:** 1899-09

**Authors:** 


Commenting upon the teeth of recruits, the *Broad Arrow*, a journal devoted to the interests of the Services, remarks: "Nearly two thousand recruits who offered themselves last year were rejected under the heading, 'loss or decay of many teeth.' This has a peculiarly local as well as general interest for the recruiting department. Lancashire lads, as well as Lancashire witches, are remarkable by the absence of the normal number of teeth, and by the lamentable disintegration of those they possess. Now, Lancashire is a very happy hunting-ground for the army —Manchester especially—and in these days when the department is at its wit's end to find the requisite supply, it might not be a bad idea to give Government aid to some local dental institution, or even to found a national dental hospital, where the lads and lasses of Lancashire could, for a nominal fee, have the necessary dentistry at their disposal. A regulation army toothbrush and a teeth-cleaning parade weekly might advantageously follow. There is no reason that the subject should not also be included in those lectures which depot officers are now urged to deliver where potential recruits abound." I think that the creation of depot dentists would be a much better plan.—*The Dental Record.*

Never Sleep.—There are several species of fish, reptiles and insects that never sleep during their stay in the world. Among fish it is now positively known that pike, salmon and goldfish never sleep at all. Also that there are several others of the fish-family that never sleep more than a few minutes a month. There are dozens of species of flies which never indulge in slumbers and from three to five species of serpents which the naturalists have never yet been able to catch napping.

According to Nottage, there is a certain variety of cold for which gelsemium is as near specific as any drug can be. The patient feels cold chills running up the back. A clear watery fluid runs from his nose. Now give the gelsemium and next morning the cold is gone.—*N. Y. Med. Times.*

